# Evaluation of Social and Physical Enrichment in Modulation of Behavioural Phenotype in C57BL/6J Female Mice

**DOI:** 10.1371/journal.pone.0024755

**Published:** 2011-09-08

**Authors:** Natalia Kulesskaya, Heikki Rauvala, Vootele Voikar

**Affiliations:** Department of Biosciences and Neuroscience Center, University of Helsinki, Helsinki, Finland; Alexander Flemming Biomedical Sciences Research Center, Greece

## Abstract

Housing conditions represent an important environmental variable playing a critical role in the assessment of mouse behaviour. In the present study the effects of isolation and nesting material on the behaviour of female C57BL/6J mice were evaluated. The mice were subjected to different rearing conditions from weaning (at the age of 3 weeks). The study groups were group- and single-housed mice, divided further into groups with or without nesting material (species-specific enrichment). After 8 weeks spent in respective conditions the behavioural testing began. Both factors (social conditions and nesting material) appeared to have a significant impact on the behavioural phenotype. However, it is important to stress that the interaction between the factors was virtually absent. We established that isolation increased locomotor activity and reduced anxiety-like behaviour in several tests of exploration. In contrast, absence of nesting material increased anxiety-like behaviour. Neither factor affected rota-rod performance, nociception and prepulse inhibition. Contextual fear memory was significantly reduced in single-housed mice, and interestingly, in mice with nesting material. Cued fear memory was reduced by single-housing, but not affected by enrichment. Mice from enriched cages displayed faster and better learning and spatial search strategy in the water maze. In contrast, isolation caused significant impairment in the water maze. In conclusion, both isolation and species-specific enrichment have profound effects on mouse behaviour and should be considered in design of the experiments and in assessment of animal welfare issues.

## Introduction

The demand for rigorous analysis of mouse behaviour has been increased tremendously. The driving force is development of functional genetics with large projects undertaken to evaluate the functions of every single gene [1]–[3]. For the analysis of new mutants the battery of behavioural tests is recommended [4], [5]. This approach should ensure that unexpected and confounding phenotypes are to be detected reliably; however, conducting the tests in the same mice in the battery might introduce special problems [6]–[8]. Along with these projects there is a considerable debate over the standardization of the behavioural experiments and environmental factors influencing behavioural data [9]–[14]. For instance, an attempt was recently made to validate the standard operating procedures in several laboratories [15], and these protocols are used for screening of mutant mice in the clinic-type approach [16]. However, again the cross-laboratory validation and role of the enrichment appeared to be a major challenge [17], [18]. On the other hand, environmental manipulations are especially useful in validation of certain scientific hypotheses (e.g. beneficial effects of enrichment, relation of stress to anxiety- and depression-like behaviour). Therefore, systematic investigation of environmental factors with possible effects on behaviour seems to be appropriate [19]–[21].

The general suggestion is to keep the mice in groups and to provide nesting material as a species-specific enrichment [22]. This is proposed to reduce the stress and increase animal welfare. However, models for stress-related disorders require application of some sort of stress in order to fulfil the validity criteria [23]. Different methods have been applied for that purpose (e.g. restraint stress, chronic mild stress, maternal separation, and social isolation). However, so far the results remain controversial. For instance, it has been shown that maternal separation in mice did not provide a model for anxiety- and depression-like behaviours [24], [25]. Recently, the use of term ‘stress’ has been challenged [26] and accordingly, it should be restricted to conditions where an environmental demand exceeds the natural regulatory capacity of an organism, in particular situations that include unpredictability and uncontrollability.

Social isolation of mice and rats is a frequently applied and investigated experimental paradigm [27], [28]. We have previously shown that social isolation in male mice has certain effects on the behaviour depending on the strain and task [29]. It has been suggested that the effects of isolation may be different in male and female mice [30]–[32] and that isolation in female mice could be a valid model for stress-related disorders [33]. Environmental enrichment has a significant impact on animal physiology [34]–[36]. However, the behavioural effects of species-specific enrichment have been addressed insufficiently [37], [38]. Therefore, the present study was designed for further clarification of isolation-induced behavioural changes in the female mice along with assessment of the role of standard nesting material as a potential modifier of these effects.

## Materials and Methods

All experiments have been carried out in accordance with the Guidelines laid down with the European Communities Council Directive of 24 November 1986 (86/609/EEC) and were approved by the County Administrative Board of Southern Finland (license number ESLH-2007-09104/Ym-23).

### Animals

The C57BL/6JOlaHsd mice were bred in the Viikki Laboratory Animal Center (University of Helsinki). Immediately after weaning (at the age of 3 weeks) the female mice were randomly allocated to four different housing conditions – a) group-housed mice with nesting material (GN); b) group-housed mice without nesting material (GNN); c) single-housed mice with nesting material (SN); d) single-housed mice without nesting material (SNN). Each group consisted of 9 mice. The behavioural experiments began when the mice were 11 weeks old. Accordingly, the mice had experienced the condition of group-housing or social isolation for 8 weeks prior to the testing. The animals were weighed weekly between 10 and 11 a.m. The bedding (aspen chips 5×5×1 mm, Tapvei Oy, Finland) was changed weekly and nesting material (aspen wool, PM90L/R, 3 mm×20 cm, Tapvei Oy, Finland) was provided as an enrichment for GN and SN mice. The food and water were available ad libitum. The animals were maintained under a 12-h light-dark cycle (lights on at 7 a.m.) at relative humidity 50–60% and room temperature 21±1°C. All experiments were carried out between 10 a.m. and 15 p.m. All behavioural tests were performed essentially as described previously [8], [29], [39] and in the order they are presented below.

### Video tracking

During the elevated plus-maze, Y-maze, and water maze tests the paths of the mice were video-tracked by using a Noldus EthoVision 3.0 system (Noldus Information Technology, Wageningen, The Netherlands). The system recorded the distance travelled by the subjects, the time spent in pre-defined zones and the status of specified event recorder keys on the keyboard. The raw data were analyzed by the same software.

### Elevated plus-maze (EPM)

EPM is a method for the assessment of unconditioned anxiety-like behaviour in rats and mice [40]. EPM consisted of two open arms (30×5 cm), two enclosed arms (30×5 cm with 15 cm high transparent side- and end-walls) and a connecting central platform (5×5 cm). The maze was raised to 38.5 cm above the floor. The mouse was placed in the center of the maze facing one of the enclosed arms and observed for 5 minutes. The following parameters were recorded by the experimenter: latency to the first open arm entry, number of open and closed arm entries and the time spent in different parts of the maze (open and closed arms, central platform). An arm entry was defined as a mouse having entered an arm of the maze with all four legs. Subsequently the percentage of the open arm visits was calculated. In addition, the number of rearings was counted.

### Light-dark exploration (LD)

The test was carried out in the open field arena (30×30 cm, Med Associates, St. Albans, VT)) equipped with infrared light sensors detecting horizontal and vertical activity. The dark insert (non-transparent for visible light) was used to divide the arena into two halves, an opening (width 5.5 cm, height 7 cm) in the wall of the insert allowed animal's free movement from one compartment to another. The light half was illuminated by 60 W light bulb 50 cm above the floor. Animal was placed in the light compartment and allowed to explore the arena for 10 minutes. Horizontal activity (distance travelled) and vertical activity (number of rearings) was recorded.

### Spontaneous activity in the open field (OF)

The mice were released in the corner of open field arena (30×30 cm, Med Associates, St. Albans, VT). Horizontal and vertical activity was recorded for one hour in 5 min intervals. Peripheral zone was defined as a 6 cm wide corridor along the wall.

### Y-maze

Spontaneous alternation performance was assessed in a symmetrical Y-maze under reduced light conditions (∼100 lx). Each arm was 30 cm long and 7 cm wide with transparent walls (15 cm high). Mice were allowed to explore the maze for 5 minutes. The number and the sequence of the arm entries were recorded. The measured variables were activity, defined as the number of arms entered, and percent alternation, calculated as the number of alternations (entries into three different arms consecutively) divided by the total possible alternations (i.e., the number of arms entered minus 2) and multiplied by 100. In addition, the number of rearings, grooming behaviour and faecal boli were recorded.

### Hot plate (HP)

Standard hot plate (TSE, Bad Homburg, Germany) was used for the assessment of nociceptive sensitivity. The plate was heated to 52°C and the mouse was confined there by plexiglass cylinder (diameter 19 cm, height 26 cm). The latency to display licking or shaking of the hindpaw was recorded.

### Rota rod (RR)

For evaluation of coordination and motor learning the accelerating rotarod (Ugo Basile, Comerio, Italy) test was performed on two consecutive days. The mice were given three trials a day with an intertrial interval of 1 hour. Acceleration speed from 4 to 40 r.p.m. over a 5-min period was chosen. The latency to fall off was the measure of motor coordination and improvement across trials was the measure of motor learning. The cut-off time was set at 6 min.

### Prepulse inhibition (PPI)

The PPI experiment was performed in Med Associates (St. Albans, VT) chambers. The isolation chambers were equipped with an acoustic stimulator and a platform with a transducer amplifier. The round acrylic holders were used for retaining the animals on the platforms. A fan and a red light were provided inside the chamber for the comfort of the animal while inside the enclosed chamber. Data acquisition was performed by using Med Associates software.

Mice were placed in the startle chamber with a background white noise of 65 dB and left undisturbed for 5 minutes. Testing was performed in 12 blocks of 5 trials and five trial types were applied. One trial type was a 40-ms, 120-dB white noise acoustic startle stimulus (SS) presented alone. In the remaining four trial types the startle stimulus was preceded by the acoustic prepulse stimulus (PPS). The 20-ms PPS were white noise bursts of 68, 72, 76 and 80 dB. The delay between onset of PPS and SS was 100 ms. The 1^st^ and 12^th^ block consisted of SS-alone trials. In remaining blocks the SS and PPS+SS trials were presented in pseudorandomized order such that each trial type was presented once within a block of 5 trials. The intertrial interval ranged between 10 and 20 seconds. The startle response was recorded for 65 ms starting with the onset of the startle stimulus. The maximum startle amplitude recorded during the 65-ms sampling window was used as the dependent variable. The startle response was averaged over 10 trials from blocks 2–11 for each trial type. The prepulse inhibition for each PPS was calculated by using the following formula: 100−[(startle response on PPS+SS trials/startle response on SS trials)×100].

### Fear conditioning (FC)

This is a form of classical conditioning where two memory components can be analysed – association of unconditioned stimulus (foot-shock, US) with a particular compartment (contextual memory) and simple association of conditioned stimulus (tone, CS) with shock. The experiments were carried out employing a computer-controlled fear conditioning system (TSE, Bad Homburg, Germany). Training was performed in a clear acrylic cage (35×20×20 cm) within a constantly illuminated (∼550 lx) fear conditioning box. A loudspeaker provided a constant, white background noise (68 dB) for 120 s followed by 10 kHz tone (CS, 75 dB, pulsed 5 Hz) for 30 s. The tone was terminated by a footshock (US, 0.7 mA, 2 s, constant current) delivered through a stainless steel floor grid (Ø 4 mm, distance 9 mm). Two CS-US pairings were separated by a 30 s pause.

Contextual memory was tested 24 h after the training. The animals were returned to the conditioning box and total time of freezing (defined as an absence of any movements for more than 3 s) was measured by infrared light barriers scanned continuously with a frequency of 10 Hz. The CS was not used during this time. Memory for the CS (tone) was tested 2 h later in a novel context. The new context was a similarly sized acrylic box. The light intensity was reduced to 100 lx, the floor was flat (without shock grid) and the background colour was black (as opposed to white colour in training context). After 120 s of free exploration in novel context the CS was applied for additional 120 s and freezing was measured as above. In addition, the activity was registered in all phases of training and testing.

### Water maze (WM)

The test was introduced for testing spatial learning and memory in rodents (Morris, 1981). The system used by us consisted of a black circular swimming pool (∅ 120 cm) and an escape platform (∅10 cm) submerged 0.5 cm under the water surface in the centre of one of four imaginary quadrants. The animals were released to swim in random positions facing the wall and the time to reach the escape platform was measured in every trial. Two training blocks consisting of three trials each were conducted daily. The interval between trials was 4–5 min and between training blocks about 5 hours. The platform remained in a constant location for 3 days (6 sessions) and was thereafter moved to the opposite quadrant for 2 days (4 sessions). The transfer tests were conducted approximately 18 h after the 6^th^ and 10^th^ training sessions. The mice were allowed to swim in the maze for 60 seconds without the platform available. The spatial memory was estimated by the time spent in the zone around the platform (∅ 30 cm) and in corresponding zones of the three remaining quadrants. In addition, the swimming distance and the thigmotaxis (wall hugging) were measured. Thigmotaxis was defined as the time spent swimming within the outermost ring of the water maze (10 cm from the wall).

After completing the spatial version of the water maze the platform was made visible in the quadrant not employed previously. The mice were tested in one block of three trials (ITI 4–5 min) and the time to reach the platform was measured.

### Forced swim test (FST)

FST is a method to estimate behavioural despair in stressful and inescapable situations [41]. The mouse was placed for 6 minutes in the glass cylinder (18 cm in diameter, 25 cm high) filled with water at 23±1°C to the height of 15 cm. The time of immobility (passive floating, when the animal was motionless or doing only slight movements with tail or one hind limb, whereas the animal was judged to be active when struggling, climbing or swimming using all four paws) was measured during last 4 minutes of the test.

### Statistics

Two-factorial ANOVA was performed with grouping (group vs single) and enrichment (nest vs no nest) as the independent variables. Post hoc comparisons were carried out by Newman-Keuls test. The repeated measures ANOVA with time or test session as a within-subjects factor was performed where appropriate (exploration tests with time intervals, water maze). Student's t-test was performed for comparing the data against the chance level in the Y-maze. Differences were considered to be significant at p<0.05.

## Results

### Body weight

**Figure 1:**
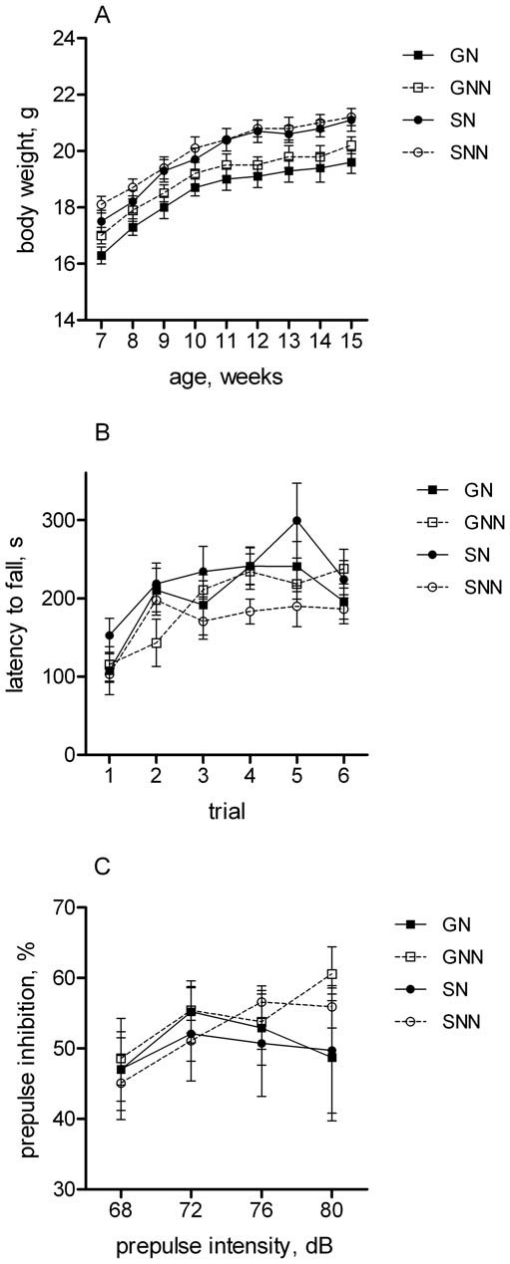
Body weight, motor and sensory functions. A) Gain of body weight: single-housed mice had increased body weight irrespective to enrichment. B) Motor learning and coordination: latency to fall from the accelerating rota-rod was not affected by different housing conditions. C) Pre-pulse inhibition: percentage of PPI at different prepulse intensities was not affected by different housing conditions.

The body weight of group-housed mice was reduced as compared to single-housed animals, irrespective of nesting material (Figure 1A). The main effect of grouping on body weight was significant [F(1,32) = 18.6, p<0.01], whereas the effect of nesting material and interaction between the factors was not significant.

### Exploratory behaviour and emotionality

For assessment of exploratory activity and emotionality a battery of tests was applied (Table 1), consisting of elevated plus maze, light-dark exploration, open field, Y-maze and forced swim test.

**Table 1:** Results of elevated plus maze (EPM), light-dark test (LD), spontaneous activity (OF), Y-maze, and forced swim test (FST).

	Group		Single		P-values (2-factorial ANOVA)		
Test and parameter	Nest(n = 9)	No nest (n = 9)	Nest(n = 9)	No nest (n = 9)	Group vs Single	Nest vs No nest	Inter-action
**EPM**							
Distance, cm	1178 (94)	1012 (62)	1552 (68)	1238 (70)	<0.01↓	<0.01↑	ns
Open arm latency, s	20.0 (3.3)	41.1 (8.3)	22.5 (4.5)	50.3 (13.3)	ns	<0.01↓	ns
Closed entries, nr	11.7 (1.2)	10.6 (0.9)	15.0 (1.1)	11.4 (1.1)	ns	<0.05↑	ns
Open entries, nr	5.2 (1.1)	3.6 (0.6)	10.8 (1.0)	5.8 (1.2)	<0.01↓	<0.01↑	ns
Open entries, %	30.5 (6.0)	25.5 (4.5)	41.5 (2.2)	32.5 (4.8)	<0.05↓	ns	ns
Open arm time, %	17.6 (7.1)	15.2 (6.3)	32.6 (2.6)	19.4 (3.4)	ns	ns	ns
Center time, %	28.7 (4.3)	33.8 (3.0)	26.5 (1.7)	33.7 (1.8)	ns	<0.05↓	ns
Rearings, nr	10.2 (1.0)	6.9 (0.6)	12.1 (2.3)	8.4 (1.1)	ns	<0.05↑	ns
**LD**							
Distance, cm	1936 (189)	2220 (104)	2695 (74)	2345 (146)	<0.01↓	ns	<0.05
Distance in light, %	38.5 (2.5)	29.9 (1.7)	43.8 (1.6)	33.3 (2.3)	<0.05↓	<0.01↑	ns
Time in light, %	33.4 (3.5)	28.0 (2.4)	47.7 (2.6)	30.8 (3.3)	<0.01↓	<0.01↑	ns
Rearings, nr	75.1 (12.0)	70.7 (4.1)	91.4 (7.1)	61.2 (6.1)	ns	<0.05↑	ns
Rearings in light, %	52.6 (3.5)	37.8 (3.1)	61.6 (3.6)	39.9 (5.1)	ns	<0.01↑	ns
**OF**							
Distance, cm	7087 (355)	6854 (530)	9780 (567)	8984 (596)	<0.01↓	ns	ns
Distance in center, %	27.6 (1.7)	25.9 (1.7)	26.6 (1.9)	21.0 (1.3)	ns	<0.05↑	ns
Time in center, %	19.2 (2.5)	15.7 (1.6)	21.9 (2.3)	12.3 (1.3)	ns	<0.01↑	ns
Rearings, nr	684.4 (71.3)	609.9 (58.5)	873.6 (65.8)	689.6 (55.7)	<0.05↓	<0.05↑	ns
**Y-maze**							
Distance, cm	1971 (200)	1947 (137)	2134 (102)	1994 (136)	ns	ns	ns
Alternation, %	57.4 (3.9)	61.7 (2.4)	60.3 (3.2)	58.0 (2.8)	ns	ns	ns
Rearings, nr	27.4 (4.6)	29.4 (3.0)	32.2 (3.1)	25.8 (2.5)	ns	ns	ns
**FST**							
Latency to float, s	48.5 (7.5)	43.6 (11.7)	81.9 (21.9)	38.4 (15.8)	ns	ns	ns
Immobility time, %	50.5 (3.7)	54.5 (4.9)	40.2 (5.9)	40.7 (6.4)	<0.05↑	ns	ns

Mean values followed by standard error of mean in parenthesis are shown for each group (ns = not significant). Arrows after significant p-values indicate direction of difference between main factors (group-housing compared to single-housing, nest compared to no-nest).

Overall, group-housed mice showed significantly reduced exploratory activity and enhanced anxiety-like behaviour as compared with single-housed mice. This was reflected in less distance travelled in the plus maze, light-dark box and open field, reduced number of open arm entries in the plus maze, reduced activity and time spent in the light compartment of light-dark box, less rearings in the open field, and increased immobility in the forced swim test (Table 1).

Nesting material as enrichment increased exploration and reduced anxiety-like behaviour in all tests as compared with animals housed in the cages without nesting material. Thus, the mice with nesting material displayed more distance travelled in the plus maze, shorter latency to enter open arm, increased number of open arm entries in the plus maze, increased activity and time spent in the light compartment of light-dark box, increased activity and time spent in the center of open field, increased number of rearings in the plus maze, light-dark box and open field (Table 1).

### Sensory and motor functions

The hot plate test was used for measuring the pain sensitivity. All groups showed similar latency to shake or lick the hindpaw (nociceptive response) when placed on the hot surface (data not shown).

Motor learning and coordination was evaluated by testing mice on the accelerating rota-rod. The repeated measures ANOVA revealed a significant effect of trial [F(5,160) = 17.2, p<0.01], indicating improvement in ability to stay and walk on the rota-rod from trial to trial (Figure 1B). However, neither main effects of grouping and nest material nor interactions between the factors were significant. Therefore, all groups improved the performance in a similar manner.

Pre-pulse inhibition of acoustic startle reflex was applied for testing sensorimotor gating. A significant main effect of pre-pulse intensity [F(3,96) = 5.5, p<0.01] confirmed that inhibition of the startle depends on the pre-pulse intensity. However, the remaining main effects (grouping, nest material) and interactions between the factors were statistically not significant, suggesting that these manipulations did not affect pre-pulse inhibition (Figure 1C).

### Learning and memory

For assessment of learning and memory we used classical fear conditioning and spatial learning in the water maze.

**Figure 2:**
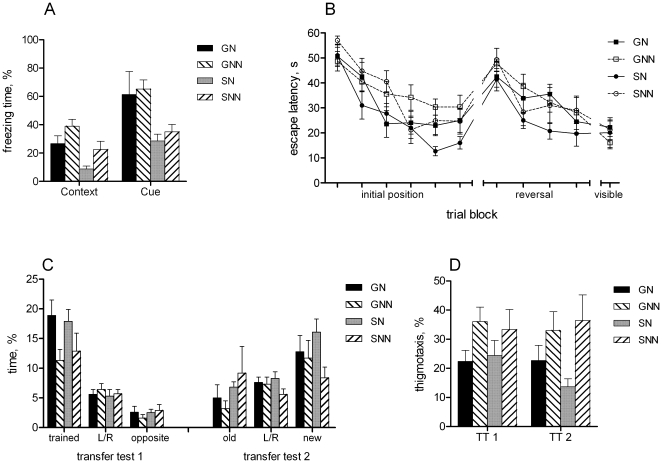
Learning and memory assessed by fear conditioning and water maze tests. A) Fear conditioning: percentage of freezing in the context and cue tests of memory 24 hours after conditioning. Single-housed mice displayed reduced freezing in both tests, animals from cages enriched with nest material showed reduced contextual freezing. B) Escape latency during learning of initial, reversed and visible platform positions. C) Percentage of time spent in the target zone and in respective zones of remaining quadrants during transfer tests. Transfer test 1: enrichment with nest increased the time spent searching at the trained zone. Transfer test 2: single-housed mice without nesting material showed no preference to any zone. D) Percentage of time in thigmotaxis during transfer tests: nesting material reduced thigmotaxis.

There was no difference in baseline freezing behaviour between the groups before training (Figure 2A). However, 24 hours after conditioning the group-housed mice showed significantly more freezing than single-housed animals when returned to the conditioning chamber [context test, main effect of grouping F(1,32) = 12.9, p<0.01]. In addition, the animals with nesting material showed less freezing than those without nest [main effect of enrichment F(1,32) = 7.3, p = 0.01]. The interaction between the factors was not significant.

When the animals were placed into novel compartment, there was again no difference in freezing between the groups (Figure 2A). However, when tone (conditioned stimulus) was applied in the novel context, the freezing of group-housed mice was significantly enhanced compared to single-housed mice [effect of grouping F(1,32) = 11.2, p<0.01]. In contrast, nesting material did not affect freezing response during the tone in novel context [effect of enrichment F(1,32) = 0.3, p = 0.59].

During initial training in water maze the animals with nests showed shorter escape latencies than animals without nesting material [Figure 2B, effect of enrichment F(1,32) = 9.1, p<0.01], suggesting enhanced learning. There was no difference between the group- and single-housed mice. A significant effect of training block [F(5,160) = 29.2, p<0.01] confirmed overall learning effect (faster finding of platform from trial to trial).

To evaluate spatial memory and search strategy the transfer tests were performed, where platform was removed from the pool. The first transfer test revealed that all groups spent more time in the target zone than in other respective zones (Figure 2C). However, it appeared that the groups with nest spent more time in the target zone than animals without nest [effect of enrichment F(1,32) = 6.9, p = 0.01]. Moreover, the nest animals displayed reduced thigmotaxis compared to no-nest animals [Figure 2D, effect of enrichment F(1,32) = 4.5, p<0.05].

The escape latencies during reversal learning (platform in opposite quadrant compared to initial location) did not differ between the groups (Figure 2B). However, the second transfer test showed that the single-housed mice without nest displayed no preference to any zone (Figure 2C), although the main effect of enrichment for the time spent in the new target zone was not significant [F(1,32) = 3.2, p = 0.08]. Similar to the first transfer test, the effect of enrichment was significant on thigmotaxis [F(1,32) = 7.1, p = 0.01], indicating reduced wall-hugging in animals with nesting material (Figure 2D).

## Discussion

In the present work we addressed the effect of group-housing and nesting material as forms of enrichment on the behavioural profile of female C57BL/6JOlaHsd mice. Our findings emphasize that both factors contribute substantially to emotional behaviour and learning and memory. Therefore, these results emphasize careful consideration of different housing conditions when behavioural studies are performed.

Investigation of the conditions that are or can potentially be stressful for mice has two sides. One aspect is the animal welfare and ethics of the research. Indeed, the problems of housing, husbandry, and handling of the laboratory animals are equally important for researchers and public opinion groups [42]–[46]. Therefore, the studies involving pain and distress are very carefully observed and discussed [47]–[50]. Moreover, the animals with violated welfare could yield inconsistent and confounded data, the number of animals needed for the experiments is larger and that definitely does not follow the generally accepted principles of 3R's for the animal experimentation [51]–[53].

On the other hand, one might be interested in the conditions that have a value for studying stress and stress-related disease models in the mice. Stress is a common experience in everyday life, but in the case of extensive stress, or inability to cope with it, severe disorders can develop in human beings. Therefore, an extensive research towards understanding the mechanisms of these disorders and relevant animal models are really needed. Many models are based on applying acute stressors, but it is clear that developmental, genetic and environmental chronic stress models could better meet the needs of research [24], [54]–[58]. However, many of these models may be considered as models of adaptation rather than models of stress-related pathology [26].

Social isolation is deleterious to health, yet little is understood about why this is so [59]. A lot of information on the behavioural effects of single housing and other sources of social stress in laboratory mice is available [28], [60]–[66]. However, there is a big variation in the duration of the isolation before the experiments (from few days to many weeks) and also in the age when the animals were isolated (immediately after weaning, during adolescence or adulthood). If we add here the variations related to genetic background and sex of the animals, conflicting results are not surprising. Moreover, whether the long-term isolation in terms of endocrine functions is truly stressful condition may remain questionable, because recent reports have shown that group-housed female mice have in fact higher corticosterone levels than single-housed mice [33], [60]. On the other hand, isolation has been shown to affect several cardiovascular and immunological parameters as a result of psychosocial stress [67], [68]. Finally, every disturbance to mouse environment can evoke autonomic stress responses [69] and accordingly, all behavioural testing, for instance, is highly stressful for animals. However, different stressors may not activate the physiological stress response to the same extent [70]. Therefore, the definition of stress is extremely crucial [26].

We selected female mice for studying the possible effects of isolation and absence of nesting material because it has been shown that females may be more sensitive than males to such environmental stressors [30]–[32], [71]. Moreover, basic studies in neuroscience use preferentially male subjects [72], [73], although women are more likely than men to suffer from depression and anxiety disorders [74], [75]. Therefore, the gender differences in susceptibility to stress-related disorders should be taken into account in the design of basic studies and animal models. The B6 mice were chosen because this is the most commonly used reference strain for behavioural phenotyping studies and for maintenance of mutant lines [76].

In neuroscience, enrichment usually means environmental stimulation that in turn could be reflected in the plasticity of nervous system and changes in the behaviour, e.g. in learning and memory, or in anxiety-like behaviour [77]. This type of enrichment is most commonly achieved by adding toys, tunnels, ladders, running wheels etc. in the home cage, and may involve regular changing of the enrichment items. It has been shown to be beneficial for mutant mice to overcome the learning deficits or to delay the onset of symptoms in the disease models [78]–[80]. However, what is the beneficial role of every piece of enrichment is often unclear [81].

In laboratory animal science, enrichment is defined as a modification in the environment that seeks to enhance physical and psychological well-being by providing stimuli meeting the animals' species-specific needs [22], [38]. Lack of appropriate enrichment can lead to maladaptive and abnormal behaviour of the animals [82]. Accordingly, both social housing and availability of the nesting material are considered to be important for mouse welfare [37], [38]. In order to better differentiate between enrichments aimed at novelty-induced stimulation (neuroscience) or at enhancement of animal welfare (laboratory animal science), it has been suggested to call the latter modifications as “environmental refinement” [38]. However, the behavioural effects of the species-specific enrichments have been addressed by far less studies than the effects of environmental stimulation.

Different housing conditions in our study played an important role in gain of body weight – single-housed mice had higher body weight than group-housed counterparts. However, nesting material appeared to have no effect on weight. Increased body weight after single housing in female mice has been shown also by others [33], whereas single housed male mice tend to have reduced body weight [29], [30], [83]. Therefore, this is already important evidence of sex difference in reaction to social isolation.

We examined the emotional behaviour of mice in three tests (elevated plus-maze, light-dark test, and activity box) that are based on the natural conflict between exploratory drive and avoidance towards unfamiliar arenas [84]. In addition, forced swim test [41] was applied for testing the coping with inescapable stress-situation, often used for screening novel antidepressants or depression-like phenotype in mutant mice [54]. Individually housed mice demonstrated reduced anxiety-like behaviour and increased locomotor activity in all three tests of anxiety-like behaviour. We have previously shown similar changes in individually housed male mice [29]. It seems to be in conflict with some other reports where reduced exploration and increased anxiety in isolated CD-1 female mice has been shown [31], [32]. On the other hand, recent studies with C57BL/6 strain did not establish any isolation-induced changes in anxiety-like behaviour either in male or female mice [60], [85]. It should be noted that in our experiment the period of isolation prior to the testing was considerably longer (8 weeks) than in the other studies (1–4 weeks). Recently, a distinction between isolation (long-term) and separation (short-term) was proposed [33], where 15 weeks of isolation resulted in mild increase of anxiety-like behaviour (assessed by light-dark test) and behavioural despair (forced swim test and tail suspension test). Increased immobility after isolation has been shown also by other groups [86]. In contrast, our experiments revealed significantly reduced immobility of individually housed mice in the forced swim test. As similar findings have been shown earlier [29], [87], it is tempting to speculate that individually housed animals could be less vulnerable to inescapable stress.

Prepulse inhibition as a model for sensorimotor gating has been studied extensively in animal models of neuropsychiatric diseases [88], [89]. Developmental models of schizophrenia have been invented, and several studies have shown deficient PPI in isolated rodents [90], [91]. However, our experiments did not reveal any effect of either isolation or nesting material on the sensorimotor gating, thus in line with some other studies where manipulations during critical developmental period have not produced defects in sensorimotor gating [25], [85].

Motor coordination and learning (assessed by accelerating rotarod) and nociception (hot plate) were not affected by different housing conditions. However, profound effects in fear conditioning and water maze tests emerged. As shown previously with male mice [29], isolation significantly reduced freezing in context and cue test of fear conditioning. Importantly, the mice with nesting material showed reduced contextual freezing. We suggest that this may be due to general reduction of anxiety-like behaviour, as revealed by conventional exploration tests. Moreover, a link between contextual fear conditioning and anxiety in mice has been shown [92]. In the water maze, the animals with nesting material displayed faster spatial learning and enhanced preference to the trained location in the probe trial. Reversal learning revealed impaired performance of single housed animals. Overall, the present data confirm earlier findings on impaired learning and memory in single housed mice [29], [86]. Regarding the nesting material, our study supports the recent finding that species-specific enrichment is beneficial for spatial learning and memory [93].

It has been shown that mice prefer conspecific housing over single-housing [94] and nesting material over barren environment [95], [96]. Both single-housing and barren environment have major impact on the behaviour of mice [82]. Previous suggestions for using nesting material as an enrichment have been based on the spontaneous preference [97] and on the fact, that nesting material has no adverse effects [98]. However, the behavioural effects of species-specific enrichments have been studied in rather limited manner [37]. Moreover, from the earlier literature it is often difficult to find the details of animal housing and husbandry or other essential information [99]. Therefore, it should be mandatory to adhere to common reporting guidelines and to include all relevant information in the publications [44]. Our experiments revealed that nesting material was an important modifier of the behavioural phenotype, as shown by significantly reduced anxiety-like behaviour of the animals that had nest material in their cages, both individually and group-housed. There was no interaction between the main factors (housing and enrichment). The effect of nesting material on emotionality was further highlighted by reduced thigmotaxis in the water maze, and reduced freezing behaviour in contextual fear test. To the best of our knowledge, this is the first study where such prominent effects of species-specific enrichment on different aspects of behaviour in laboratory mice are shown.

### Conclusion

In general, the results of our experiments showed that social isolation of female C57BL/6J mice for at least 8 weeks from weaning resulted in increased body weight, enhanced explorative activity and stress tolerance. In contrast, lack of nesting material produced substantial increase in anxiety-like behaviour. Learning and memory was negatively affected by both single housing and lack of nesting material. Therefore, we conclude that lack of environmental stimulation (both physical and social) has profound effects on mouse behaviour. This knowledge could be helpful for design and interpretation of stress-related animal models and is relevant to be considered for animal welfare issues.
